# Congenital Zika Virus Infection in a Birth Cohort in Vietnam, 2017–2018

**DOI:** 10.4269/ajtmh.20-0286

**Published:** 2020-08-17

**Authors:** Mya Myat Ngwe Tun, Masako Moriuchi, Michiko Toizumi, Elizabeth Luvai, Sandra Raini, Noriko Kitamura, Mizuki Takegata, Hien-Anh Thi Nguyen, Meng Ling Moi, Corazon C. Buerano, Dang Duc Anh, Lay-Myint Yoshida, Kouichi Morita, Hiroyuki Moriuchi

**Affiliations:** 1 Department of Virology, Institute of Tropical Medicine and Leading Program, Nagasaki University, Nagasaki, Japan;; 2 Department of Pediatrics, Graduate School of Biomedical Sciences, Nagasaki University, Nagasaki, Japan;; 3 Department of Pediatric Infectious Diseases, Institute of Tropical Medicine, Nagasaki University, Nagasaki, Japan;; 4 National Institute of Hygiene and Epidemiology, Hanoi, Vietnam;; 5 Research and Biotechnology, St. Luke’s Medical Center, Quezon City, Philippines

## Abstract

To detect congenital ZIKV infection (CZI) in a birth cohort and among high-risk neonates in Vietnam, we collected umbilical cord blood plasma samples of newly delivered babies and peripheral plasma samples of high-risk neonates in Nha Trang, central Vietnam, between July 2017 and September 2018. Samples were subjected to serological and molecular tests. Of the 2013 newly delivered babies, 21 (1%) were positive for Zika virus (ZIKV) IgM and 1,599 (79%) for *Flavivirus* IgG. Among the 21 ZIKV IgM-positives, 11 were confirmed to have CZI because their plasma samples had anti-ZIKV neutralization titers ≥ 4 times higher than those against dengue virus (DENV)-1 to 4 and Japanese encephalitis virus (JEV) and were tested for the ZIKV RNA positive by real-time reverse transcription–PCR. Therefore, the incidence of CZI in our birth cohort was approximately 0.5%. Of the 150 high-risk neonates, three (2%) and 95 (63%) were positive for ZIKV IgM and *Flavivirus* IgG antibodies, respectively. None of the three ZIKV IgM-positives had ≥ 4 times higher anti-ZIKV neutralization titers than those against DENV-1 to 4 and JEV, and were therefore considered as probable CZI. Our results indicate that CZI is not rare in Vietnam. Although those with confirmed CZI did not show apparent symptoms suspected of congenital Zika syndrome at birth, detailed examinations and follow-up studies are needed to clarify the CZI impact in Vietnam. This is the first report of CZI cases in a birth cohort in Asia.

## INTRODUCTION

Zika virus (ZIKV) of the family Flaviviridae, genus *Flavivirus* can be transmitted to humans through the vector *Aedes* mosquitoes or through nonvector transmission such as sexual contact, maternal–fetal transmission, and blood transfusions.^1–5^ The first human case of ZIKV infection was reported in 1954 in Nigeria, and sporadic cases have been noted in Asia.^6,7^ It has been widely reported that approximately 80% of people with ZIKV infection are asymptomatic.^8,9^ Although the disease is self-limiting, cases of neurological manifestations have been described. Between 2015 and 2016, ZIKV had been of global health concern following large outbreaks in the Americas and the observed associated congenital abnormalities, including microcephaly, intrauterine growth restriction, blindness, and stillbirth.^10^ Despite a long period of ZIKV circulation in Asia, only three confirmed cases of congenital ZIKV infection (CZI) with microcephaly were reported in this region: two in Thailand and one in Vietnam.^11,12^ In Vietnam, 219 and 13 cases of ZIKV disease were reported in 2016 and 2017 (January–February), respectively.^13^ No data are available on the incidence and embryotoxicity of CZI in a birth cohort in Asia. We herein report data of ZIKV infection from 1) a large-scale birth cohort study on mother-to-child infections and 2) investigation of neonates who were suspected with congenital infection in Vietnam.

## MATERIALS AND METHODS

### Study participants and sample collection.

The present study was conducted in Khanh Hoa General Hospital (KHGH), Nha Trang, Vietnam, from July 2017 to September 2018, and consisted of two parts. For the first part of the study, we enrolled all women who 1) delivered their babies at KHGH, 2) were 18 years or older at the time of delivery, and 3) resided in selected 16 communes in Nha Trang, during the study period. Exclusion criteria for this part of the study were women who had 1) spontaneous/induced abortions or stillbirths, 2) multiple pregnancies, or 3) serious complication from/during this pregnancy. Blood samples were collected from umbilical cords of babies just after their delivery at the obstetrics ward. ethylenediaminetetraacetic acid-treated tubes were used for blood collection. Plasma was separated by centrifugation (3,000 rpm × 10 minutes) and kept in a −80°C freezer until testing.

For the second part of the study, during the study period, we enrolled high-risk neonates (children aged 28 days or less) 1) born at KHGH from women who had any two disease symptoms such as fever, rash, arthralgia/arthritis, lymphadenopathy, and conjunctivitis, or 2) born at KHGH or referred to neonate intensive care unit/pediatric department in KHGH and who had any symptoms related to congenital infection such as suspected meningoencephalitis, microcephaly, hydrocephalus, glaucoma, cataract, thrombocytopenia, purpura, hearing impairment, and lymphadenopathy, or who had head circumference of < 30 cm at birth, and whose birth weight for gestation age was equal or below the cutoff on birth weight patterns by gestation age reference setting.^14,15^ Exclusion criteria for the second part were neonates with confirmed chromosomal abnormality or those with well-known congenital syndrome related to a genetic defect. Some of the participants in the second part of the study could also be included in the first part if their mothers lived in the catchment area and delivered them at KHGH during the study period. Peripheral blood samples were collected from the neonates, and the plasma was separated and kept as earlier.

Written informed consents were obtained from mothers for their participation in the first part of the study as well as for their babies’ participation in the first and/or second parts of the study.

This work was approved by the Ethics Committees of the Institute of Tropical Medicine, Nagasaki University, Japan (160908158), and the National Institute of Hygiene and Epidemiology, Hanoi, Vietnam (IRB-VN01057-30/2015).

### Viruses and cell lines.

The virus strains used for the serological tests, namely, IgM capture ELISA, Flavi IgG ELISA, and neutralization tests, were as follows: MR 766 (ZIKV), 99St12A (dengue virus [DENV]-1), 00st22A (DENV-2), SLMC50 (DENV-3), SLMC318 (DENV-4), and JaOrS982 (Japanese encephalitis virus [JEV]). These viruses were propagated in C6/36 *Aedes albopictus* mosquito cells and were used to inoculate the Vero (African green monkey kidney epithelial cell line ATCC, CCL81) cell line for virus titration and neutralization tests.

### IgM capture ELISAs for the detection of ZIKV, DENV, and JEV infections.

In-house IgM capture ELISAs were carried out using the protocol described previously with minor modifications.^16^ Each well of 96-well microplates (Maxisorp, Nalge Nunc International, Roskilde, Denmark) was coated with 100 μL (5.5 μg/100 μL) of antihuman IgM goat IgG antibody (Cappel ICN Pharmaceuticals, Aurora, OH) in ELISA coating buffer (0.05 M carbonate–bicarbonate buffer, pH 9.6, containing 0.0% sodium azide). Plates were then incubated at 37°C for 1 hour or at 4°C overnight. Each well was blocked with 100 μL of BlockAce (UK-1 B 80, Yukijirushi, Sapporo, Japan), except for the blank wells, and plates were incubated at room temperature for 1 hour. After incubation, wells were washed three times with phosphate-buffered saline (PBS) without calcium and magnesium but containing 0.1% Tween 20 (PBS-Tween 20 [PBS-T]). Test samples, as well as positive and negative control samples, were diluted at 1:100 in PBS-T, and 100 μL aliquots of these samples were distributed into duplicate wells. Plates were incubated at 37°C for 1 hour and then washed as described earlier. Zika virus or tetravalent DENV or JEV antigen (128 ELISA units) at 100 μL/well was added after which the plates were incubated at 37°C for 1 hour. After washing as described earlier, horseradish peroxidase (HRP)-conjugated anti-*Flavivirus* mouse monoclonal antibody (12D11/7E8) at 1:1,000 dilution for anti-ZIKV IgM capture ELISA, or 1:1,500 dilution for anti-DENV or anti-JEV IgM capture ELISA was added at 100 μL/well. Plates were incubated at 37°C for 1 hour and washed as earlier. Color was developed by adding in each well a 100-μL volume of 5 mg *o-*phenylenediamine dihydrochloride (OPD) (Sigma Chemical, St. Louis, MO) with 0.0% hydrogen peroxide in 10 mL of 0.05 M citrate phosphate buffer, pH 5.0. Plates were kept at room temperature for 30–60 minutes in a dark place. To terminate the reaction, 100 μL of 1 N sulfuric acid was added to each well, and then the optical density (OD) was read at 492 nm (Multiscan JX, model no. 353; Thermolab System, Tokyo, Japan). A positive control (or test sample)/negative control OD ratio greater than or equal to 2.0 was considered positive.

### Anti-*Flavivirus* IgG ELISA.

In-house indirect IgG ELISA was performed to detect the presence of anti-*Flavivirus* IgG in plasma samples, and a purified JEV was used as assay antigen.^17^ In the procedure, all wells of the 96-well microplates, except for the blanks, were coated with 100 µL of JEV antigen (250 ng/100 µL/well) diluted with ELISA-coating buffer. Plates were incubated at 37°C for 1 hour or at 4°C overnight. All wells except for the blanks were blocked with 100 µL of the original concentration of BlockAce and were incubated at room temperature for 1 hour. Plates were washed three times with PBS-T, after which 100 µL of each test plasma sample diluted at 1:1,000 in PBS-T + 10% BlockAce was added in duplicate wells in each plate. Control sample known to contain the antibody to test antigen was run on each plate as a positive control. After incubation at 37°C for 1 hour, plates were washed, and 1:30,000 diluted HRP-conjugated antihuman IgG goat IgG (American Qualex, San Clemente, CA) in PBS-T + 10% BlockAce was added at 100 µL/well. Plates were incubated at 37°C for 1 hour, followed by washing. Initiation of the peroxidase reaction was performed by the addition of OPD substrate solution (described earlier) at 100 µL/well. Plates were incubated at room temperature for 30–60 minutes in the dark, and then the reaction was stopped by the addition of 1 N sulfuric acid at 100 µL/well. A standard curve was prepared by using the OD_492_ values of the positive control serum starting with a 1,000-fold dilution, followed by serial 2-fold dilutions up to 1:2^12^ in PBS-T + 10% BlockAce. IgG titers of test serum samples were determined from the positive standard curve. A sample titer equal to or greater than 1:3,000 was considered to be positive.

### Focus reduction neutralization test.

To confirm the status of ZIKV infection in the study subjects, plasma samples were checked for the ability to neutralize ZIKV, the four serotypes of DENV, and JEV by 50% focus reduction neutralization test (FRNT_50_).^16,18^ Plasma samples were heat-treated at 56°C for 30 minutes and diluted serially. Serially diluted samples at 150-µL volumes were mixed with equal volumes of specific virus at 60 focus-forming units, and mixtures were incubated at 37°C for 1 hour for virus–antibody neutralization reaction. Each mixture was inoculated onto Vero cell monolayer in 96-well plates. After incubation at 37°C for 1 hour, the infected cells were overlaid with 1.3% methylcellulose 4,000 in 2% fetal calf serum minimum essential media. The plates with ZIKV or JEV were then incubated at 37°C for 2 days, and the plates with DENV were incubated for 3 days in the same temperature. The plates were washed with PBS (−). Cells in each plate were fixed with 4% paraformaldehyde phosphate-buffered solution for 30 minutes at room temperature, rinsed, and were permeabilized with 1% NP-40 solution in PBS (−) for 30 minutes at room temperature. After washing, the plates were blocked with BlockAce for 30 minutes at room temperature. Pooled human serum samples with a high titer of anti-*Flavivirus* IgG (diluted 1:1,500) were then added per well; plates were then incubated at 37°C for 1 hour and washed. Subsequently, 1:1,000 diluted HRP-conjugated goat antihuman IgG was added to each well, after which plates were incubated at 37°C for 1 hour. Staining of positive cells was visualized by the addition of a 0.5-mg/mL solution of substrate 3, 3′-diaminobenzidine tetrahydrochloride in PBS (−) with 0.0% of H_2_O_2_ at room temperature. Staining reaction was allowed to proceed for 10 minutes, after which the cells were washed. The number of foci of stained cells per well was counted under a microscope. The reciprocal of the endpoint serum dilution that provided a 50% or greater reduction in the mean number of foci relative to the control wells that contained no serum was considered to be the FRNT_50_ titer. The volume used for all the reactants in this test was at 100 µL/well.

### Conventional and real-time reverse transcription–PCR (qRT-PCR) for ZIKV.

Viral RNA was directly extracted from plasma by using Viral RNA Mini Kit (QIAGEN, Hilden, Germany) according to the manufacturer’s instructions. The qRT-PCR was performed by using TaqMan Fast Virus 1-Step Master Mix kit (Applied Biosystems, Foster City, CA) following the protocol from a previous report with three primer sets for ZIKVE and NS5 genes.^19–23^ Cycle threshold value < 40 was considered as ZIKV positive for qRT-PCR. Standard complementary DNA at 10-fold serial dilutions (10^8^–10^2^ genome copies) was applied for quantification of viral genome levels.^20^ The viral genome levels were expressed as log_10_ genome copies/mL. Conventional RT-PCR was performed by using Prime Script one step RT-PCR Kit (Takara Bio Inc., Shiga, Japan) following the manufacturer’s instructions and using previously published primers for ZIKVNS3 gene.^24^

### Zika virus case classification.

We regarded a case as CZI if ZIKV RNA was detected in the cord or neonatal blood plasma or if the neonate was ZIKV IgM positive and positive for neutralization test only against ZIKV but not with other flaviviruses or when the neutralizing antibody titer against ZIKV was ≥ 4 times higher than the antibody titers against other flaviviruses. This definition is consistent with confirmed ZIKV infection in the WHO criteria.^25^ The WHO defined a ZIKV infection as a probable case if the clinical sample is positive for IgM antibody against ZIKV and the ratio of ZIKV neutralization titer to other *Flavivirus* neutralization titers is less than 4 and with no ZIKV RNA detected by RT-PCR.^25^ In case there is no adequate plasma sample for the performance of RT-PCR, we also considered infection as probable. In this study, we called our probable case as probable CZI and regarded it as non-confirmed CZI.

## RESULTS

In total, 2015 mothers, who were about to give birth, were enrolled in the first part of the study, and a total of 2013 umbilical cord plasma samples from their newly born babies were analyzed. In the second part of the study, 150 neonates were enrolled, and their plasma samples were also analyzed. In the first part of the study, 21 (1%) of the 2013 newly born babies were positive for ZIKV IgM, seven (0.3%) for DENV IgM, and 1,597 (79.3%) for *Flavivirus* IgG (Table 1). Among the 21 ZIKV IgM-positives, nine were positive for JEV and/or DENV IgM. In the second part of the study, of the 150 neonates, three (2%), two (1.3%), and 95 (63.3%) were positive for ZIKV IgM, DENV IgM, and *Flavivirus* IgG, respectively (Table 1). Of the three ZIKV IgM-positives, one was JEV IgM positive.

**Table 1:** Positive rates of ZIKV IgM, DENV IgM, and *Flavivirus* IgG in pregnant women and neonates, Nha Trang, Vietnam, July 2017–September 2018

Source of samples	Total no. of samples	ELISA-positive samples		
		ZIKV IgM	DENV IgM	Flavi IgG
Cord blood plasma in the birth cohort	2,013	21 (1.0%)	7 (0.3%)	1,597 (79.3%)
Plasma from high-risk neonates	150	3 (2.0%)	2 (1.3%)	95 (63.3%)

DENV = dengue virus; ZIKV = Zika virus.

Among the 21 ZIKV IgM-positive newly born babies, 11 had anti-ZIKV neutralization titers ≥ 4 times higher the neutralization titers against DENV-1 to 4 and JEV (Table 2). Therefore, 11 (0.5%) in the birth cohort were confirmed as CZI. In addition, their samples had ZIKV IgM *P*/*N* ratios higher than those for DENV and JEV. Six of the 11 babies had plasma ZIKV RNAs that were positive in the real-time PCR in the two primer sets used in the study, whereas five were positive in one of the two primer sets. However, detection of their ZIKV RNAs by conventional RT-PCR showed negative results. The other 10 of 21 ZIKV IgM-positives had cord blood plasma samples with anti-ZIKV neutralization titers < 4 times than those against DENV-1 to 4 and JEV, and thus were considered to have probable CZI.

**Table 2:** Confirmation of Congenital ZIKV infection in the birth cohort, Nha Trang, Vietnam, July 2017–September 2018

			Gestational age (weeks/days)	Birth weight (g)	Head circumference (cm)	Maternal age (years)	IgM			*Flavivirus* IgG titer	Neutralization titer (50% focus reduction neutralization test)						ZIKV real-time reverse transcription–PCR (copies/mL)	
Study year	No.	Gender					ZIKV	DENV	JEV		ZIKV	JEV	DENV-1	DENV-2	DENV-3	DENV-4	E gene primer	NS5 gene primer
2017	1	F	38/0	2,800	32.5	34	**2**	0.7	0.6	41,712	**1,280**	160	80	160	160	320	2.8 × 10^4^	1.8 × 10^4^
	2	M	38/2	3,000	34.5	34	**3.3**	0.7	0.1	8,518	**640**	< 80	< 80	80	< 80	< 80	UND	3.2 × 10^4^
	3	M	39/3	3,400	33	38	**3.7**	0.8	0.3	29,541	**640**	80	160	160	160	< 80	4.1 × 10^4^	2.1 × 10^4^
	4	F	40/1	3,100	33	29	**3.7**	2.5	2.3	72,232	**1,280**	80	320	320	320	320	UND	6.5 × 10^4^
	5	M	40/4	3,100	33.5	29	**2**	0.6	0.5	17,318	**640**	< 80	80	160	< 80	80	1.3 × 10^4^	UND
	6	F	40/2	3,600	34.3	23	**2**	0.8	0.5	31,677	**2,560**	80	640	640	160	320	1.3 × 10^3^	UND
	7	M	40/2	2,800	32	28	**17.9**	14.9	13.1	72,944	**20,480**	160	5,120	5,120	5,120	1,280	2.2 × 10^4^	UND
	8	F	39/3	3,400	33.5	42	**7.9**	6	5.8	57,360	**2,560**	320	160	320	< 80	640	4.0 × 10^4^	1.2 × 10^4^
	9	F	39/2	3,100	33	21	**8.6**	2	0.6	10,320	**1,280**	160	320	160	160	320	1.6 × 10^4^	3.5 × 10^4^
2018	10	M	39/6	4,000	34.5	28	**2**	1.4	0.5	33,395	**320**	80	80	< 80	80	80	1.9 × 10^3^	UND
	11	F	37/2	2,500	31.5	25	**2.3**	0.6	1.2	24,035	**640**	< 80	80	160	160	80	2.6 × 10^3^	1.3 × 10^4^

DENV = dengue virus; F = female; JEV = Japanese encephalitis virus; M = male; UND = undetermined; ZIKV = Zika virus; cutoff value of IgM *P*/*N* ratio = 2; cutoff value of IgG titer = 3,000.

ZIKV IgM and neutralization titers, which are diagnostically important, are shown in bold figures

The three ZIKV IgM-positive neonates in the second part of the study had plasma samples with anti-ZIKV neutralization titers < 4 times than those against DENV-1 to 4 and JEV. They were considered as probable CZI and hence were not confirmed CZI. With regard to annual and seasonal patterns for CZI, there were nine confirmed cases of CZI in July–November 2017 and two in July–September 2018, suggesting annual variability and seasonality. Our findings showed approximately 0.5% incidence of CZI, or 5.5 per 1,000 live births in our birth cohort study in central Vietnam.

Among the 11 confirmed CZI cases, nine babies did not show any apparent symptoms at birth and were discharged together with their mothers after 5 days as usual delivery hospitalization. One baby girl who showed symptoms of illness vomited repeatedly and did not suck her mother’s breast milk on the next day after birth. Her white blood cell count was 20,110/µL (neutrophil 67.7%). She was hospitalized in the pediatric ward, treated with antibiotics for neonatal infection, and given phototherapy for neonatal jaundice. She was discharged after 5 days of hospitalization. The other baby girl who showed symptoms of illness was born to a mother who had pre-labor rupture of membranes. The baby, too, had vomited and could not be breastfed. Her white blood cell count was 28,300/µL (neutrophil 71.0%) on the day of birth. She was treated with antibiotics for neonatal bacterial infection and had phototherapy for neonatal jaundice at the pediatric department for 11 days. After getting well, she was discharged. These two baby girls did not show any symptoms suspected as congenital infection as listed in the inclusion criteria for the second part of the study, and they were not enrolled in the second part. The 10 cases with probable CZI in the first part of the study had no apparent symptoms at birth.

## DISCUSSION

In this study, we reported the occurrence of CZI among babies delivered in Nha Trang, Vietnam. Their infections were confirmed based on the positive detection of ZIKV RNA, IgM against ZIKV, and neutralizing antibodies against ZIKV by using their cord blood plasma samples. It was noted that these samples had positive results for IgM and/or neutralization activity against more than one virus. This could be because of cross-reaction of the tested antibodies against ZIKV, DENV, and JEV, all of which belong to the same Flaviviridae family.^26–28^ High percentage of cord blood and neonate plasma samples positive for anti-*Flavivirus* IgG was observed, and it could be because of the passively transferred maternal IgG.^29^ Increasing number of divergent ZIKV strains that highlight genetic variability is regarded as a potential limiting factor of the sensitivity of ZIKV qRT-PCR based diagnosis; therefore, a previous study suggested to use several qRT-PCR targets for diagnosis.^23^ The 11 babies with confirmed CZI in this study had their plasma samples positive for qRT-PCR in at least one primer set. However, conventional PCR performed to plasma positives by qRT-PCR showed negative results which could be because of low viral loads in the plasma. Previous studies reported that conventional RT-PCR could detect ZIKV viral load in most of the samples, with an estimated 10^6^–10^10^ RNA copies/mL.^23,30^

Published studies showed strong association between microcephaly and ZIKV infection confirmed by qRT-PCR, capture IgM ELISA, or both.^21,31,32^ In Brazil, 32 of 91 neonates born with microcephaly were confirmed positive for ZIKV infection by qRT-PCR or anti-ZIKV IgM ELISA with confirmation more frequent in cerebrospinal fluid than in serum.^33^ Also in Brazil, another study reported that levels of ZIKV IgM and neutralizing antibodies were higher in babies with microcephaly cases than in the neonate controls (at the time of birth) and their mothers.^34^ In our study, we confirmed CZI in 0.5% (11/2013) of newly born babies based on the results of IgM capture ELISA, qRT-PCR, and neutralization test by using their cord blood samples. Comparing our data from the Americas where the rate of microcephaly/CZI varies from 5% to 14%,^35,36^ our study showed a low rate of CZI infection, and it could be associated with the differences in the characteristics of the ZIKV belonging to the different clades of Asian lineage. A previous study on the importation of ZIKV from Vietnam to Japan in 2016 indicated that the isolated virus belonged to the Southeast Asian clade of the Asian lineage, and it was distinct from the ZIKV isolates (American clade of Asian lineage) in the Americas.^37^ Other reports suggested that Southeast Asian clade of ZIKV had lower replicative ability than the American clade of Asian lineage/African lineage.^38^

This is the first report of ZIKV infection in a birth cohort in Asia. Our results indicate that CZI is not rare in Vietnam. Even though eye examinations and brain imaging were not conducted in this study, all the infected babies did not show apparent symptoms suspected of congenital Zika syndrome, which are characterized by severe microcephaly (in which the skull has partially collapsed), decreased brain tissue with a specific pattern of brain damage, damage to the back of the eye, congenital contractures, and hypertonia restricting body movement soon after birth.^39,40^ Newborns whose mothers are infected with ZIKV during pregnancy have a 5–14% risk of congenital Zika syndrome and a 4–6% risk of ZIKV-associated microcephaly,^35,36,41–46^ whereas a study involving pregnant women from Rio de Janeiro used a broader definition for ZIKV-associated outcomes and identified adverse outcomes in 42% of fetuses and infants exposed to the virus.^41^ Thus, generally, the number of children who were born to mothers with ZIKV infection during pregnancy but who did not have apparent disability at birth is large, and our findings are in agreement with that. However, a previous study found that infants with in utero ZIKV exposure without congenital Zika syndrome appeared at risk for abnormal neurodevelopmental outcomes in the first 18 months of life.^39^ Therefore, detailed clinical assessment combined with ophthalmologic examination, hearing screening, and brain imaging, and long-term follow-up including neurodevelopmental surveillance of infected offspring are needed to clarify the impact of CZI in Vietnam.
